# Use it or lose it: a four-year follow-up assessing whether physical activity near one’s capacity reduces the risk of functional decline among older adults

**DOI:** 10.1186/s11556-025-00385-8

**Published:** 2025-10-27

**Authors:** Antti Löppönen, Katja Lindeman, Lotta Palmberg, Evelien Van Roie, Christophe Delecluse, Erja Portegijs, Taina Rantanen, Timo Rantalainen, Laura Karavirta

**Affiliations:** 1 University of Jyväskylä, Jyvaskyla, Finland; 2 University of Turku, Turku, Finland; 3 Hasselt University, Hasselt, Belgium; 4 KU Leuven, Leuven, Belgium; 5 KU Leuven, Leuven, Belgium; 6 University of Groningen, Groningen, Netherlands

**Keywords:** Device-based, Mobility limitation, Free-living activities, Walking test

## Abstract

**Background:**

Physical capacity (PC) defines the limits for physical activity (PA), while activities in daily life typically remain submaximal. Older adults whose daily activities approach their physical capacity may experience less functional decline, though prospective evidence is limited. This study compared changes in physical function over a four-year follow-up between community-dwelling older adults categorized based on their combined baseline physical capacity and physical activity.

**Methods:**

312 community-dwelling older adults (75–85 years, 60% women) participated in this study. Baseline physical capacity was measured using the 5-second Mean Amplitude Deviation (MAD) during a maximal 10-meter walking test. Physical activity was assessed based on individuals’ ~99.25th percentile MAD values from free-living accelerometry (representing an intensity equivalent to 75 min/week of physical activity), which were then used for group categorization into lowPC-lowPA, lowPC-highPA, highPC-lowPA, and highPC-highPA profiles. Physical function was assessed with the Short Physical Performance Battery (SPPB) and the 5x Sit-To-Stand (5xSTS) test. Analyses used nonparametric tests and generalized estimating equations.

**Results:**

Significant changes in SPPB and 5xSTS were observed in all profiles (*p* < 0.05) except for the lowPC-highPA profile. The decline in SPPB was greater for low versus high physical activity profiles in both PC profiles (high PC: B -0.61, SE 0.24, 95% CI -1.08, -0.15; low PC: B -0.96, SE 0.35, 95% CI -1.62, -0.32), but no significant difference was found for the decline in 5xSTS time between physical activity profiles in either physical capacity profile.

**Conclusions:**

Engaging in physically demanding activities, irrespective of baseline physical capacity, may help slow functional decline in old age. Older adults should be encouraged to engage in physically demanding activities to enhance their functional capacity.

**Supplementary Information:**

The online version contains supplementary material available at 10.1186/s11556-025-00385-8.

## Background

Physical functioning has been identified as an important factor that enables independent living among older adults [1]. The most effective way to maintain physical functioning is through diverse and sufficiently challenging physical activity and exercise, which should also include activities tailored to the individual’s capabilities [2]. However, multiple internal and external factors, such as ability to walk and social support, affect physical behaviour resulting in considerable variation in daily physical activity between older adults [3, 4].

Physical activity has been presented as a distinct construct from physical capacity [5, 6]. Although physical capacity and physical activity are correlated, they represent distinct constructs. Physical capacity primarily defines the limits of physical activity rather than ensuring that individuals use their full capacity in daily life [5, 6]. According to the mechanobiology of the locomotory system, disuse leads to atrophy while increased use promotes hypertrophy [7]. Therefore, if full functional capacity is rarely utilised, it is likely to decline, capturing the essence of the ‘use it or lose it’ principle. This demarcation between activity and capacity and the associated use-dependent adaptations have been operationalized in the physical capacity-physical activity (PC-PA) concept proposed by Koolen et al. and Orme et al. which categorizes individuals into four profiles: low PC - low PA (“cannot do – does not do”), low PC - high PA (“cannot do, does do”), high PC - low PA (“can do, does not do”) and high PC - high PA (“can do, does do”) [8, 9].

To our knowledge, no studies have focused on PC-PA profiles in older adults in a longitudinal setting, which allows prediction of future conditions based on the profiles. Additionally, the methods used to determine PC and PA have not been “apples-to-apples” comparisons. Rather, the operationalization has been, e.g., PC determined by walking distance or the Timed Up and Go (TUG) compared with PA estimated by daily step counts [8, 10, 11]. Orme and colleagues introduced in their technical note that PC-PA profiles can be created by instrumenting a walking test and free-living PA with an accelerometer [9], thereby ensuring a direct “apples-to-apples” comparison of the intensity of PC and PA using acceleration.

We propose addressing PA and PC intensities from free-living and standardized testing, respectively, using 5-second Mean Amplitude Deviation (MAD) epochs [12]. This approach allows for the examination of the PA intensity distribution, which can be used to determine the vigorous MAD intensity threshold - the MAD value that corresponds to the 75 minutes per week of physical activity recommended by the World Health Organization (WHO) [13]. As such, it is well-suited for defining ‘does do, does not do’ in PA. This is meaningfully comparable to PC, which was defined as the MAD during a 10-meter walking test [14].

So far, there are no prospective studies shedding light on whether older people who approach their capacity in free-living activities have a reduced risk of future decline in physical function. Therefore, the aim of this study was to compare changes in physical function over four years between community-dwelling older adults categorized on the basis of their baseline PC and PA. We hypothesized that those older adults who were physically active close to their physical capacity would experience a slower decline in physical function.

## Methods

### Participants and design

The data for this observational study were drawn from data collected in the AGNES study (Active Aging - Resilience and external support as modifiers of the disablement outcome; n = 1 021), which was conducted at the Gerontology Research Center, University of Jyväskylä.. The AGNES study comprises three age cohorts (75, 80, and 85 years of age) of people living independently in the city of Jyväskylä, in Central Finland. The baseline data were collected in 2017–2018 and the 4-year follow-up measurements were carried out in 2021–2022. The Ethical Committee of the Central Finland Health Care District provided an ethical statement on the research plan and protocol of the AGNES baseline (August 23, 2017) and follow-up study (September 8, 2021). The study was executed in accordance with the principles of the Declaration of Helsinki and all participants gave written informed consent.

Baseline recruitment was drawn as a random sample from postcode areas in Jyväskylä, Finland, using the registers of the Digital and Population Data Services Agency in Finland. Baseline inclusion criteria were age and residence in the study area, willingness to participate and the ability to communicate [15]. After exclusions, 1021 individuals participated in the study, of whom 432 wore a tri-axial accelerometer for 3 to 7 consecutive days and participated in the 10-meter walking test with an accelerometer, forming a baseline sample for this study. Of the older adults included in the baseline sample, 26 were deceased, 6 could not be reached, 77 were not interested or not applicable, and 11 had not completed the Short Physical Performance Battery (SPPB) at the time of the follow-up, resulting in a final follow-up sample of 312 participants. All of these participants had at least 3 days of successful accelerometer recording and completed the maximal 10-meter walking test at baseline, and also participated in the 4-year follow-up home interview with the SPPB test.

### Determination of PC-PA profiles

**Fig. 1:**
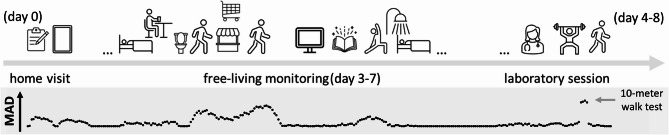
Timeline of study protocol and typical occurrence of 5-second MAD epochs

At baseline, a research assistant visited the participant’s home, conducted a face-to-face interview, and placed an accelerometer on the participant’s thigh using a waterproof film. Participants wore the accelerometer continuously (24 h a day) for 3 to 7 consecutive days. Subsequently, participants attended a laboratory visit where they underwent a comprehensive health and physical function assessment, which included a maximal 10-meter walking test (Fig. 1).

The study employed a UKK RM42 tri-axial accelerometer (13-bit analog-to-digital conversion, acceleration range ± 16 g, UKK Terveyspalvelut Oy, Tampere, Finland). The accelerometer sampling rate was set at 100 samples per second, with acceleration recorded in gravity units. From the data collected by the accelerometer, the mean amplitude deviation (MAD = 1/n *∑ |rk –r|) of each 24-hour period was calculated based on the vector magnitude (Euclidian norm) of the resultant acceleration (√x2 + y2 + z2) in non-overlapping 5-second epochs, following the methodology outlined in previous reports [12].

**Fig. 2:**
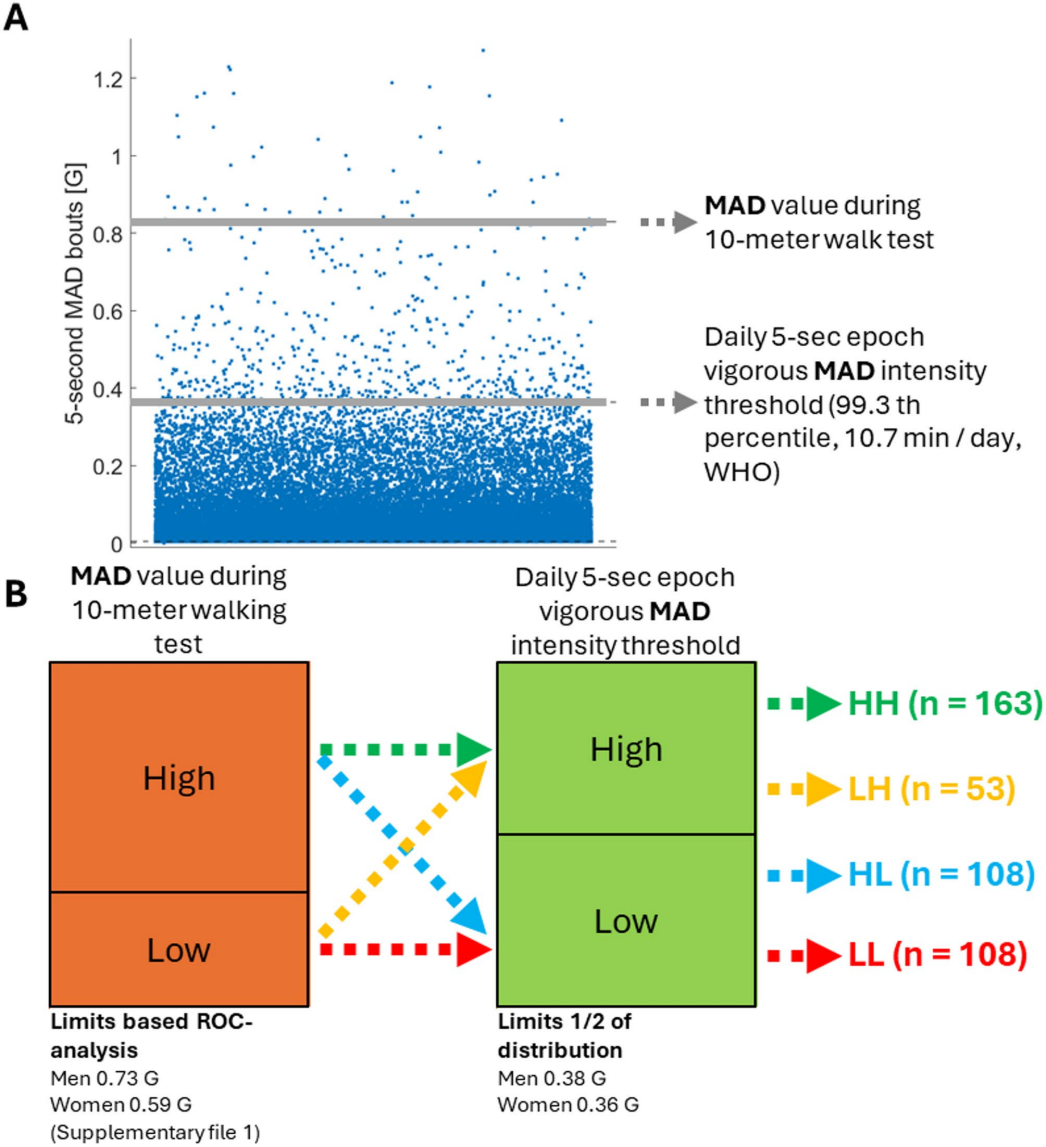
**A** Specification of the variables used for PC-PA profiling. **B** Generating PC-PA profiles

 Laboratory-based assessment of physical capacity (PC) and determination of ‘Can do’ and ‘Cannot do’ groups Physical capacity (PC) was determined based on the mean amplitude deviation (MAD) epoch during the maximal 10-meter walking test [16] (Fig. 2A), which was part of a structured and time-controlled laboratory protocol. The test was conducted in a research laboratory walking track, with a total distance of 20 m reserved for the test. The test area included a 5-meter acceleration phase, a 10-meter test section, and a 5-meter deceleration phase. During the maximal 10-meter walking test, participants were instructed to walk safely from the starting point to the end point at their maximum walking speed. The representative MAD value was taken from the 5-second epoch exhibiting the highest vector magnitude during the identified 10-meter test phase. The MAD value during 10-meter walking test was strongly correlated with maximum walking speed of same test in this dataset (*n* = 432, *r* = 0.72, *p* < 0.001).

To the best of the authors’ knowledge, there are no thresholds for the MAD value during the maximal 10-meter walking test for this age group (75–85 years), based on which capacity can be classified as non-limited and limited physical functioning [17]. Therefore, in this study, the thresholds for the MAD value during 10-meter walking test were determined separately for men and women using Receiver Operating Characteristics (ROC) in classifying the dataset into high (SPPB ≥ 10) and low (SPPB < 10) physical functioning [18]. For men, a cut-point of 0.73 G was determined with moderate accuracy (specificity 70%, sensitivity 66%, AUC 0.76), and similarly, for women, the cut-point was 0.59 G (specificity 68%, sensitivity 76%, AUC 0.73). More detailed analysis can be found in Supplementary File 1.

#### Physical activity (PA) assessment and the determination of ‘Does do’ and ‘Does not do’ groups

The ‘Does do’ and ‘Does not do’ groups were defined based on the entire free-living monitoring period (aggregating full 24-hour measurement days) using the 5-second MAD epoch distribution (intensity profile), which describes the dispersion of activity intensities. The highest part of the distribution represents the highest attained intensity [19] (Fig. 2A). The individual peak 75-min MAD intensity threshold of the 5-second MAD epoch distribution was determined to represent the threshold for 75 minutes per week (10.7 minutes per day). The 75-minute threshold is based on the physical activity guidelines by the WHO [13] as the recommended weekly amount of vigorous activity. This threshold represents the ~ 99.25 percentile of the activity intensity distribution reflecting the intensity of the most active minutes of the week for each individual. The 75-minute duration was chosen to capture a meaningful volume of high-intensity activity that could plausibly reflect physical effort near one’s capacity, in line with the health-promoting potential highlighted in the WHO guidelines. It indicates that the activity intensity exceeds this level only ~ 0.75% of the time each day, which equates to 10.7 minutes out of the total 1440 minutes in a day. The ‘Does do’ and ‘Does not do’ groups were defined via a data-driven approach by dividing the participants into two equally sized groups based on a median split of the individual peak 75-minutes MAD intensity thresholds, calculated separately for men and women (Fig. 2B). As part of the sensitivity analyses, we also tested alternative thresholds for physical activity (PA), including a 105-minute weekly cut-off. These analyses did not result in notable changes in the distribution of individuals across the PA and PC profiles, indicating that the profile classification remained stable regardless of the threshold used.

#### Generation of PC-PA profiles

Finally, the PC and PA categories were combined to create four profiles: LL = low PC – low PA (“cannot do – does not do”); LH = low PC – high PA (“cannot do, does do”); HL = high PC – low PA (“can do, does not do”); and HH = high PC – high PA (“can do, does do”) following the approach presented by Koolen et al. and Orme et al. [8, 9] (Fig. 2B).

### Lower extremity functioning as a follow-up outcome

For the longitudinal analyses, lower extremity functioning was assessed at baseline and follow-up in the participants’ homes using the Short Physical Performance Battery (SPPB). The SPPB comprised tests of standing balance, walking speed over a 3-meter distance, and the five-times-sit-to-stand (5xSTS) test [20, 21]. In this study, we used the SPPB total score (maximum of 12 points, with higher scores indicating better lower extremity functioning) and the time in seconds of the 5xSTS test as outcomes.

### Descriptive characteristics and other measurements

Age and sex were obtained from the population register and cognitive function was assessed using standardized procedures (Mini-Mental State Examination, MMSE [22]).

*Walking difficulties* over distances of 500 m and 2 km were investigated by asking the participants, “Do you have difficulty walking 2 kilometres/500 meters?” The response options included: (1) able to manage without difficulty, (2) able to manage with some difficulty, (3) able to manage with a great deal of difficulty, (4) able to manage only with the help of another person, and (5) unable to manage even with help. In this study, response options 2–5 were grouped into the category “I have walking difficulties,” with response option 1 indicating “I do not have walking difficulties” [23, 24].

*Self-rated health status* was assessed with the question: “How would you rate your current overall health?” The response options were: “1. excellent”, “2. good”, “3. fair”, “4. poor”, “5. very poor”. For the analysis, response options 1 and 2 were categorized as “good perceived health” and response options 3–5 were combined into the category “limited perceived health”. *The perceived ability to perform desired activities* from the perspective of health was assessed with the question: “To what extent has your health or physical ability prevented you from doing the things you wanted to do in the past 4 weeks?” The response options were: “1. not at all”, “2. a little”, “3. somewhat”, “4. a lot”, “5. extremely.” For the analysis, response option 1 was categorized as “my health does not prevent me from doing things I want” and response options 2–5 were combined into the category “my health prevents me from doing things I want” [25].

*Maximal isometric knee extension strength* was assessed in the laboratory (at a knee angle of 60 degrees from the fully extended leg to flexion) of the dominant leg in a seated position using an adjustable dynamometer chair (Metitur LTD, Jyväskylä, Finland). At least three attempts were required, and the highest force (N) was chosen for the analysis [26]. *Maximal isometric handgrip strength* was measured on the dominant side with a hand-held adjustable dynamometer (Jamar Plus digital hand dynamometer, Patterson Medical, 6 Cedarburg, WI, USA) and expressed in kg [27].

### Statistical analyses

Baseline comparisons between the different profiles were made across the entire sample of 432 older adults. Baseline descriptive data are presented as means and standard deviations (SD) for continuous variables and relative frequencies (%) for dichotomous variables. Due to some skewness in the data and variability in sample sizes of the profiles, non-parametric tests were used to compare baseline characteristics. Differences in baseline characteristics between PC-PA profiles were tested by independent samples Kruskal-Wallis test for continuous variables and by Chi-square test for dichotomous variables. Pairwise comparisons between profiles were Bonferroni-corrected.

Changes over four years in SPPB scores and 5xSTS test time were examined for a longitudinal sample (*n* = 312) who had data from both baseline and follow-up measurements. The Wilcoxon signed rank test for related samples was used to analyze changes within the different PC-PA profiles in the total SPPB score and in the 5xSTS test time. For the Wilcoxon signed-rank test, effect sizes were calculated using the formula r = Z/√N, where Z is the standardized test statistic and N is the number of paired samples.

Generalized estimating equations (GEE) [28] with a linear link function and unstructured working correlation matrix were used to assess differences in the SPPB total score and the 5xSTS test time between low and high PA profiles (group effect), and to evaluate how these outcomes change over time across groups (group x time interaction). GEE was chosen because it accounts for within-subject correlation in repeated measures and provides robust, population-averaged estimates, which align with the study’s aim of comparing average physical function trajectories between groups, rather than focusing on individual-specific changes. Since only two time points were included, an unstructured working correlation matrix was selected to maximize flexibility in modeling the correlation between repeated measures. To mitigate potential misspecification of the working correlation structure and ensure valid inference, robust (sandwich) standard errors were employed. Analyses were performed separately for low PC and high PC profiles. All GEE models were adjusted for sex, age cohort, self-rated health status, days included in the accelerometry analysis and 10 m MAD. Sex, age, and self-rated health were included as covariates because they may affect physical capacity and physical activity [29–31]. The number of valid measurement days was included to account for the opportunity for activities to occur (e.g., number of walking bouts). The 10-meter MAD was included to adjust for baseline physical capacity. Population-averaged coefficients (B), standard errors (SE), and 95% confidence intervals are reported. Statistical significance was set at *p* < 0.05, and statistical analyses were performed using the SPSS statistical software package (IBM Corp. Released 2021. IBM SPSS Statistics for Windows, version 28.0. Armonk, NY: IBM Corp.) [32]. Figures were generated in the “R” statistical environment (version 4.3.1) [33].

## Results

### Baseline characteristics of the generated PC-PA profiles

The descriptive baseline characteristics of the PC-PA profiles for the entire sample of 432 older adults are presented in Table 1. According to the baseline characteristics, the low PC profiles differed from each other in terms of 10-meter walking speed, SPPB total score, self-reported walking difficulty at 500 m and 2 km distances, self-rated health status and self-reported limited ability to perform desired activities. The high PC profiles differed from each other in terms of age and self-reported walking difficulty in 500 m and 2 km distances (Table 1).

**Table 1:** Baseline characteristics according to the PC-PA profiles (*n* = 432)

	LL low PC-low PA (*n* = 108)	LH low PC-high PA (*n* = 53)	HL high PC-low PA (*n* = 108)	HH high PC-high PA (*n* = 163)	*p*-value
Women	58%	49%	61%	63%	0.321 †
Age	79.1 (3.9)	77.6 (3.2)	78.9 (3.3)	77.6 (3.1)	**0.001** ‡ ^LL-HH, HL-HH^
MMSE	27.2 (2.6)	26.9 (2.6)	27.4 (2.2)	28.0 (1.9)	**0.005** ‡ ^LH-HH^
Leg strength [N]	302 (109)	345 (110)	348 (108)	372 (110)	**< 0.001** ‡ ^LL-HL, LL-HH^
Grip strenght [kg]	30.3 (10.7)	34.2 (12.4)	32.4 (10.5)	33.3 (10.7)	0.076 ‡
10-meter maximal walking speed [m/s]	1.44 (0.29)	1.75 (0.26)	1.90 (0.33)	2.00 (0.32)	**< 0.001** ‡ ^LL-LH, LL-HL, LL-HH, LH-HH^
5xSTS time [s]	14.3 (4.3)	12.8 (3.6)	11.4 (3.2)	11.7 (2.9)	**< 0.001** ‡ ^LL-HL, LL-HH^
SPPB total score	9.4 (2.1)	10.2 (1.8)	10.9 (1.3)	11.0 (1.2)	**< 0.001** ‡ ^LL-LH, LL-HL, LL-HH^
SR walk difficulty 500 m	36%	9%	16%	3%	**< 0.001 **† ^LL-LH, LL-HL, LL-HH, HL-HH^
SR walk difficulty 2 km	54%	15%	27%	7%	**< 0.001 **† ^LL-LH, LL-HL, LL-HH^
SR health status is weak	71%	40%	42%	37%	**< 0.001 **† ^LL-LH, LL-HL, LL-HH^
SR limited ability to perform desired activities	61%	25%	37%	23%	**< 0.001 **† ^LL-LH, LL-HL, LL-HH^

*MMSE *Mini-Mental State Examination, *STS S*it-to-stand transitions, *SPPB *Short Physical Performance Battery, *SR *Self- rated

Bold font indicates statistical significance (*p* < 0.05)

† Chi-square test and pairwise comparison with Bonferroni correction

^‡^ Independent-samples Kruskal-Wallis test and pairwise comparison with Bonferroni correction

**Fig. 3:**
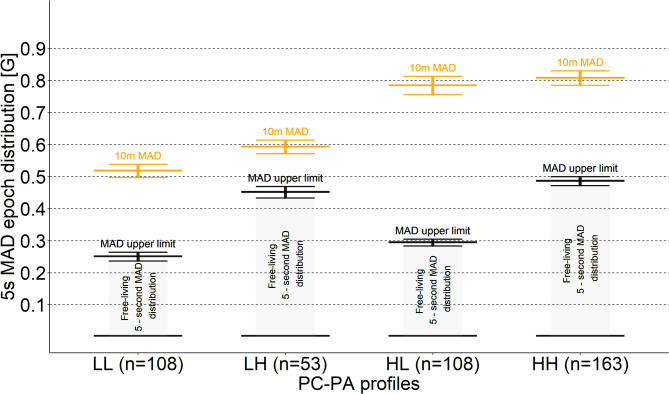
Distribution of the different PC-PA profiles according to the MAD value *MAD value during the 10-meter walking test (highlighted in orange) and the peak 75-minutes MAD intensity threshold (~ 99.25 percentile) (shown in black)*

Figure 3 shows the baseline intensity distribution of PC-PA profiles together with their MAD values during the laboratory-based 10-meter maximal walking test (highlighted in orange) and the distribution of MAD values, including their vigorous MAD intensity threshold (shown in black), for free-living 5-second MAD epochs. According to the figure, the capacity of the HL (high PC-low PA) and HH (high PC-high PA) profiles does not differ statistically (confidence intervals overlap), whereas the physical activity differs significantly between these profiles.

### Changes in the SPPB total score and the 5xSTS time in a four-year follow-up

A longitudinal sample (*n* = 312) of those who participated in both baseline and follow-up measurements was used to examine the difference in change in the SPPB total score and the 5xSTS time within and between the different profiles over four years of follow-up. During follow-up, the total SPPB score decreased by at least 2 points in 43% of participants in the low PC - low PA profile, 18% of participants in the low PC - high PA profile, 29% of participants in the high PC - low PA profile, and 15% of participants in the high PC – high PA profile. The change in the SPPB total score and the 5xSTS test time within the different PC-PA profiles is shown in Table 2. From baseline to follow-up, statistically significant changes in SPPB total score and 5xSTS test time were observed in all other profile than in the low PC – high PA profile.

**Table 2:** Changes within the different PC-PA profiles in SPPB total score and 5xSTS test time over four years of follow-up (*n* = 312)

	LL low PC-low PA (*n* = 65)	LH low PC-high PA (*n* = 39)	HL high PC-low PA (*n* = 86)	HH high PC-high PA (*n* = 122)
	Mean (SD)	Mean (SD)	Mean (SD)	Mean (SD)
BL SPPB total score	9.7 (2.0)	10.5 (1.7)	11.0 (1.3)	10.9 (1.2)
FU SPPB total score	8.3 (2.7)	10.1 (1.7)	10.1 (2.2)	10.6 (1.5)
SPPB total score difference	−1.4 (2.0)	−0.4 (1.6)	−0.9 (1.9)	−0.3 (1.4)
† *p-value*	**< 0.001**	0.059	**< 0.001**	**0.013**
* effect size	− 0.57	− 0.30	− 0.47	− 0.22
	Mean (SD)	Mean (SD)	Mean (SD)	Mean (SD)
BL 5xSTS test time [s]	13.6 (3.8)	12.3 (3.3)	11.2 (2.8)	11.7 (3.0)
FU 5xSTS test time [s]	15.7 (4.8)	13.1 (3.3)	12.8 (4.3)	12.3 (3.4)
5xSTS test time difference [s]	+ 2.0 (5.0)	+ 0.7 (3.2)	+ 1.6 (4.1)	+ 0.6 (3.2)
† *p-value*	**0.002**	0.137	**0.005**	**0.033**
* effect size	− 0.38	− 0.24	− 0.31	− 0.19

*BL *Baseline 2017–2018, *FU *Follow-up 2021–2022, *SPPB *Short Physical Performance Battery, *5xSTS *Five-times-sit-to-stand test

Bold font indicates statistical significance (*p* < 0.05)

† Wilcoxon signed rank test for related samples

* Effect size (r) was calculated as Z/√N, where Z is the standardized test statistic and N is the number of paired observations

The results of the covariate-adjusted GEE models between high and low PA profiles, separately for low and high PC profiles, are presented in Table 3. Baseline level of the SPPB total score or 5xSTS test time did not differ between low and high PA profiles in either PC profile. Over the follow-up, the decrease in SPPB total score was greater for low PA profiles compared to high PA profiles in both PC profiles (high PC: *p* = 0.010, low PC: *p* = 0.006). For the 5xSTS test, the difference in change in test time between the low and high PA profiles did not reach statistical significance for either PC profile (high PC: *p* = 0.058, low PC: *p* = 0.107).

**Table 3:** GEE model estimates for group effect and group-by-time interactions for SBBP total score and 5xSTS test time (*n* = 312)

	**SPPB total score**				**5xSTS test time**			
	Group effect		Group x Time		Group effect		Group x Time	
	B (SE)	95% CI	B (SE)	95% CI	B (SE)	95% CI	B (SE)	95 % CI
**HH **	Ref.				Ref.			
**HL**	-0.07 (0.17)	-0.41, 0.27	**-0.61 (0.24)**	**-1.08, -0.15**	-0.30 (0.38)	-1.03, 0.43	1.0 (0.53)	-0.04, 2.03
	Group effect		Group x Time		Group effect		Group x Time	
	B (SE)	95% CI	B (SE)		B (SE)	95% CI	B (SE)	95 % CI
**LH**	Ref.							
**LL**	-0.17 (0.35)	-0.87, 0.52	**-0.96 (0.35)**		0.10 (0.65)	-1.18, 1.38	1.30 (0.81)	-0.28, 2.88

All GEE models are adjusted for sex, age, self-reported health status, 10m MAD and days included in accelerometer analysis. Bold font indicates statistical significance (*p* < 0.05)

*SPPB *Short Physical Performance Battery, *5xSTS test *Five-times-sit-to-stand test, *B *The population-averaged coefficient, *SE *Standard error, *95% CI *95% confidence interval, *HH *High PC - high PA, *HL *High PC - low PA, *LH *Low PC - high PA, *LL *Low PC - low PA

## Discussion

To increase our understanding of the effects of challenging one’s abilities for maintaining physical functioning in old age, this study compared changes in physical function over 4-year follow-up among community-dwelling older adults categorized based on their baseline physical capacity and physical activity. The changes observed from the baseline to the follow-up within the different profiles demonstrated a statistically significant change in the SPPB total score and 5xSTS test time in all profiles, with the exception of the low PC-high PA profile. Over the follow-up period, the decrease in the SPPB total score was deeper for low compared to high PA profiles in both PC profiles. However, no statistically significant difference was observed in the change in 5xSTS test time between the low and high PA profiles for either PC profile. Based on the changes within the profiles, our results suggest that ageing reduces lower extremity function in older adults regardless of their physical capacity or intensity of physical activity, but by challenging themselves to be active close to their capacity, it is possible to slow the decline in lower extremity function. Our results also suggest that older adults who already have limitations in physical function can maintain their level of functioning through physical activity.

In this study, the profiles were formed based on the profiling presented by Koolen and colleagues [8]. The lower capacity profiles showed statistically significant differences in terms of walking speed, 5xSTS test time, and maximum knee extension strength in the descriptive data. However, in the two higher capacity profiles, no statistical differences were observed in functional ability and capacity variables. The descriptive profiles are consistent with the findings of Adams and colleagues [10], where the timed “up and go” (TUG) test, used as a basis for capacity assessment, showed differences among the lower capacity profiles, unlike in the higher capacity profiles. To better understand the decline in functional capacity from the perspective of the “use it or lose it” principle [34], this study focused particularly on the intensity profile of daily activities The 5-second MAD epochs provided a valuable method for capturing wide variety of intensities intensity, although the epoch length may not fully account for the most intense activities, such as jumps. Nonetheless, they offer sufficient representation, and compared to 1-second epochs, they avoid capturing brief impacts that do not reflect actual activity patterns. Significant changes in detecting higher intensities are observed with epochs of 10 s or longer [35, 36]. Although the use of the same MAD metric for both physical capacity and physical activity allows for more direct comparisons, the contexts and underlying constructs differ. Physical capacity is assessed under standardized maximal test conditions, whereas physical activity reflects free-living behavior, which is influenced by individual choice, environmental context, and daily variation. This distinction highlights the complementary nature of these two constructs despite the shared measurement scale.

In the study, a discrepancy was observed between the SPPB and 5xSTS results. This may be because the total SPPB score is a combination of gait speed, balance, and the 5xSTS test, thus providing a broader picture of functional decline. The 5xSTS result may reflect that device-based methods cannot capture activities such as strength and power training and therefore cannot distinguish activities that specifically influence 5xSTS performance.

This study was not specifically designed to investigate why some individuals utilize their capacity while others do not. However, previous research suggests that walking is one of the most popular forms of physical activity among older adults [37]. Brisk walking and other hobbies including high-intensity activities (ball games, jogging, aerobic exercise), may help explain why participants in the high physical activity (PA) profiles accumulated more activity closer to their capacity compared to those in the low PA profiles. It can therefore be assumed that sports activities play a significant role in explaining why some people consistently engage in activities that approach in terms of intensity their physical capacity. However, this is undoubtedly related to psychological and environmental factors and personal preferences [38–41]. Future research should explore these factors to design targeted interventions that promote physical activity. Additionally, based on this study, it can be inferred that even if physical capacity is already reduced, it is important to approach it habitually in order to maintain or at least slow down the decline in physical function. Obviously, safety should be considered to prevent adverse events, such as falls, or other medical emergencies potentially associated with vigorous activity.

When evaluating the results of the study, several limitations and strengths need to be considered. In this study, PC-PA profiles were constructed using a data-driven approach, consistent with previously proposed categorization methods [8, 9, 11]. One limitation of this approach is the potential loss of granularity, as any form of grouping inevitably reduces the detail in the data. Nevertheless, defining PC-PA profiles in this way can provide meaningful insights that support more personalized and targeted strategies for promoting physical activity among older adults. This study aims to address the well-known limitation that accelerometers cannot detect activities like swimming, cycling, carrying a child, walking uphill, or carrying a load [42] by examining the intensity distribution across all 5-second MAD bouts (Fig. 2), which provides a broader understanding of activity patterns. In addition, the study includes self-reported measures, such as health status, which may introduce subjectivity and reporting bias. Finally, younger and healthier participants were more likely to attend follow-up, potentially leading to limiting generalizability to the oldest-old or those with poorer physical function [43]. The strength of the study lies in sufficient sample size of independently living older adults and its longitudinal design. Furthermore, the study benefits from the continuous device-based measurement of physical behaviour for multiple days [44, 45] and employing a like-for-like capacity and free-living physical behaviour assessment.

## Conclusions

The findings suggest that engaging in physical activity (PA) close to one’s personal capacity (PC) may help protect against functional decline among older adults. The study also indicates that even older adults with existing functional limitations can slow the deterioration of their physical abilities through physical activity in a free-living environment. Therefore, older adults should be encouraged to safely challenge themselves with physically demanding activities that could potentially maintain physical function. A key question that arises is why some older adults, particularly those in good physical condition, do not challenge their functional capacity. This issue warrants further investigation in future studies.

## Supplementary Information

Supplementary Material 1.

## Data Availability

After completion of the study, data will be stored at the Finnish Social Science Data Archive without potential identifiers (open access). Until then, pseudonymized datasets are available to external collaborators subject to agreement on the terms of data use and publication of results. To request the data, please contact Professor Taina Rantanen (taina.rantanen@jyu.fi).

## Abbreviations

MAD: Mean Amplitude Deviation
PA: Physical activity
PC: Physical capacity
SPPB: Short Physical Performance Battery
STS: Sit-to-stand
